# 

**Published:** 1850-07

**Authors:** 


### INDEX, V. 10.

PAGE.

A

Amalgam, observations on the use of . . . . . 22
Amalgam, Evans' new 51, 126
Aberration in the position of a tooth, singular, . . . . 54
Antagonizing instruments for artificial teeth, . . . . 54
Annual meeting of the Mississippi Valley Association of Dental Surgeons, fifth, . . . 55
American Institute, Fair of the 58
Adjustment of Clasps, . . . 99
Arkansas Oil Stones, . . . 134
Accupuncturation in Facial Neuralgia, . . . . . 135
Artificial Nose and Palate, case of, . . . . . . 165
Antagonizing Inst'ment, Evan's, 206
American Society of Dental Surgeons, Tenth Annual Meeting of, . . . . . . 208
American Med. Formulary, . 222
Alumni, Address to the Society of the, . . . . 180, 225
American Society of Dental Surgeons, Ninth Ann. Meeting of, 293
Antagonizing, cost single, . 29

B

Baltimore College of Dental Surgery, Diploma of, . . . 139
Baltimore College of Dental Surgery, Ann. commencement of, 211
Bonnhorst, C. Von, Letter from, . . . . . . 223
Brown, Dr. Solyman, Circular of, . . . . . . 224

PAGE.

Blandy on Diseases of the Gums, &c., . . . . . 290
Brewster, Dr. C. S., . . 308

C

Cushman, Dr. C. T., Letter from, . . . . . 29, 61
Cone, Dr. C. O., Letter from, . 54
Castings, Metallic, . . . 58
Compound Screw Forceps, . 79
Cavity Plates, Lateral, 106, 108
Correspondents, to, . . . 140
Case of Artificial Nose and Palate, . . . . . 165
Court Patronage, and Professional Jealousy in England, 217
Chloroform, use of, in Dental Surgery, . . . . . 221
Clasps, Adjustment of, . . 99
Clasps, Temporary Fastenings for, . . . . . . 34
Compound Screw Forceps, improvement in, . . . . 78
Completion of Vol. X., . . 309

D

Dayton, Dr. A. C., Letter from, 42
Dentist's Lathe, . . . . 53
Dental Operations, a System of Recording by Indicators, . 74
Dry Cupping to relieve Tooth-Ache, . . . . . . 135
Diploma of the Balt. College of Dental Surgery, . . . 139
Dental Medicine, . . . 139
Dental Drills, . . . . . 205
Dental Surgeons,
Ninth Annual meeting of the American Society of, 293
Tenth Annual Meeting, . 208

PAGE.
Dentition, Paralysis which attends, . . . . . 265
Defective Utterance, peculiar case of, . . . . . 274
Disease of the Gums and Exfoliation of the Alveolo Processes, &c., . . . . . 290
Dental Ethics, . . . 79

E

Evans' new Amalgam, . 51, 126
Elliott, Dr. W. H., Letter from, 78
Ethics, Dental, . . . . 79
England, Professional Jealousy in, . . . . . . . 140
Evans' Antagonizing Instrument, . . . . . . 208
Errata, . . . . . . 224

F

Fifth Annual Meeting of the Mississippi Valley Association of Dental Surgeons, . 55
Fair of the American Institute, 58
Forceps, Compound Screw, . 79
Filling Teeth, Evans' Amalgam for, . . . . . . 126
Filling Teeth after the Pulps has Suppurated, . . . . . 162
Fogle's Single Antagonizing Cast, . . . . . . . 29
Fogle's Masticating Blocks, . 32
Fogle's Temporary Fastenings for Clasps, . . . . . 34
Fusible Metals, . . . . 60

G

Gums, case of Ulceration and Suppuration of the, . . 290
Gold, Lumps of, . . . 134
Gold Foil, . . . . . 308

H

Harris's Dental Surgery, . . 309
Human Teeth, Researches on the Developement and Structure of the, . . . . . 140
Highly Creditable, . . . 142
Handy, Dr. W. R., Letter from, . . . . 143, 216

I

Imposter, an . . . . . 140

PAGE.
J

Jealousy, Professional, in England, . . . . . 140, 217

L

Letters from
C. T. Cushman, M. D. 29, 61, 144
Dr. A. C. Dayton, . . 42
C. O. Cone, M. D., . . . 54
Dr. W. H. Elliott, . . 78
Dr. W. R. Handy, . 143, 216
Jeremiah Mason, . . . 222
C. Von Bonnhorst, . . 223
G. Y. Shirley, . . . 289
Our Paris Correspondent, . 304
Lathe, new Dentist's, . . 55
Lateral Cavity Plates, . 106, 108
Lumps of Gold, . . . 134

M

Meeting of Alumni of Balt. Col., 306
Mouth, pathological relations of the, . . . . . . . 3
Metallic Castings, . . . 58
Memoir of the late Alexander Nasmyth, . . . . . 149
Maxillary bone, resection of the left superior, . . . 172
Mechanical Dentistry, application of Tortoise shell to, . 199
Mason Jeremiah, Letter from . 222
Medical Journal contemplated, new, . . . . . . 224
Mineral Teeth, . . . . 283
Masticating blocks, . . . 32
Metastasis of the Menstrual secretion, . . . . . 42
Muscle, new, . . . . 143

N

Neuralgia facial, accupuncturation in, . . . . . 135
Nasmyth Dr. Alex., remarks on the late, by Chapin A. Harris, M. D., . . . . . 145
Memoir of the late, . . 149
Nose and Palate, case of artificial 165
Neuralgia vs. Tooth Ache, . 169
New Muscle, . . . . 143

O

Observations on the use of amalgam, . . . . . . 22
Oil Stone, Arkansas, . . 134

PAGE.
Opening Address, delivered before the Society of the Alumni of the Balt. Col. Dent. Surg., 180
Omitted articles, . . . . 224
Observations on the transplantation of Teeth, . . . . 64
Observations on the notice of, . 257

P

Pathological Relations of the Mouth, . . . . . 3
Plugging, Treatment of Dental pulp preparatory to, . . . 44
Practical Hints, No. 2, . . . 136
Professional jealousy in England, 140
Paralysis which attends Dentition, . . . . . . . 265
Peculiar case of Defective Utterance, . . . . . . 274
Paris Correspondent, our, . 304
Plates, raised, method of stiffening, . . . . . . 41

R

Researches on the Developement and Structure of the Human Teeth, . . . . 140, 223
Resection of the left Superior Maxillary Bone, . . . 172
Robinson, James, Address before Soc. Alumni of Balt. Col., 225
Rodrigues, Dr. B. A., . . 308

PAGE.

S

Singular Aberration in the position of a Tooth, . . . . 54
System for Recording Dental Operations, . . . . . 74
Speculum Oris, . . 223
Shirley, G. Y., Letter from, . 289

T

Townsend's, Dr. Address, . 309
Treatment of Dental Pulp preparatory to Plugging, . . 44
Tooth, Singular Aberration in the position of a, . . . 54
Transplantation of Teeth, . 64
Tooth-Ache, Dry Cupping to relieve, . . . . . . 135
Tortoise Shell, application of, to Mechanical Dentistry, . . 199
Table of the Contraction of Metals, . . . . . . 59

U

Use of Chloroform in Dental Surgery, . . . . 221
Utterance, peculiar case of Defective, . . . . 274

V

Valedictory Address, . . 309

